# The Emerging Role of Tertiary Lymphoid Structures in Breast Cancer: A Narrative Review

**DOI:** 10.3390/cancers16020396

**Published:** 2024-01-17

**Authors:** Dana Narvaez, Jorge Nadal, Adrian Nervo, María Victoria Costanzo, Claudio Paletta, Fernando E. Petracci, Sergio Rivero, Alexis Ostinelli, Berenice Freile, Diego Enrico, Maria Teresa Pombo, Mora Amat, Edgar Danilo Aguirre, Matias Chacon, Federico Waisberg

**Affiliations:** Breast Cancer Division, Alexander Fleming Institute, Buenos Aires 1425, Argentina; jorgecnadal@gmail.com (J.N.); nervoadrian@yahoo.com (A.N.); mariavictoriacostanzo@gmail.com (M.V.C.); cpaletta@alexanderfleming.org (C.P.); fernandopetracci@gmail.com (F.E.P.); sergio.rivero84@gmail.com (S.R.); alexisostinelli@gmail.com (A.O.); berefreile@gmail.com (B.F.); diego-enrico@hotmail.com (D.E.); mteresapombo@hotmail.com (M.T.P.); moraamatpat@gmail.com (M.A.); daniloaguirre@gmail.com (E.D.A.); matiemi@yahoo.com (M.C.); fwaisberg12@gmail.com (F.W.)

**Keywords:** breast cancer, immunotherapy, tertiary lymphoid structures, lymphocytes, prognostic factors, biomarkers

## Abstract

**Simple Summary:**

This narrative review discusses the role of tertiary lymphoid structures (TLSs) in breast cancer. TLSs are ectopic lymphoid formations that can develop in response to inflammatory signals. They are present in about 60% of breast cancer cases and are particularly common in the triple-negative subtype. TLS presence is linked to better outcomes, and they could serve as prognostic and predictive markers for treatment response. Additionally, TLS-positive tumors may show improved outcomes regardless of other factors like PDL-1 expression or TILs. The emergence of TLS as a potential therapeutic avenue highlights the importance of standardized pathology methods for their detection in cancer research.

**Abstract:**

This narrative review aims to clarify the role of tertiary lymphoid structures in breast cancer. We examine their development, composition, and prognostic value, and current ways of recognizing them. A comprehensive literature review was performed using the PubMed/Medline, Scopus, and EMBASE databases. A significant area of interest in breast cancer research involves targeting immune checkpoint molecules, particularly in the triple-negative subtype, where treatment options remain limited. However, existing biomarkers have limitations in accurately predicting treatment response. In this context, tertiary lymphoid structures (TLSs) emerge as a prognostic biomarker and also as a promising predictive marker for response. TLSs are ectopic lymphoid formations or neo-organogenesis that can develop after prolonged exposure to inflammatory signals mediated by chemokines and cytokines. Their presence is inversely correlated with estrogen receptor (ER) and/or progesterone receptor (PR) expression, but positively associated with a higher pathologic complete response rate and improved overall survival. In certain scenarios, TLS-positive tumors were associated with improved outcomes regardless of the presence of PDL-1 (programmed cell death ligand 1) expression or TILs (tumor-infiltrating lymphocytes).

## 1. Introduction

Immunotherapy currently serves as the cornerstone of new drug treatments for various types of cancer. In the case of breast cancer, immunotherapy has become a standard treatment for both advanced and early stages of the disease, specifically targeting certain patient subgroups, like the triple-negative subtype [1]. However, existing biomarkers have limitations in accurately predicting treatment response. In fact, the efficacy of immune checkpoint inhibitors in the neoadjuvant setting remains unaffected by PDL-1 expression, which is the most common marker used to define the activity of these agents in different tumor models [2,3].

Tertiary lymphoid structures (TLSs) are ectopic lymphoid formations that can develop after prolonged exposure to inflammatory signals mediated by chemokines and cytokines. These structures bear resemblance to secondary lymphoid structures (SLSs) and usually include germinal centers surrounded by T lymphocytes, dendritic cells, and high endothelial venules (HEVs) in close proximity [1,2]. TLSs, unlike SLSs, lack a surrounding capsule and are typically observed in the context of chronic non-neoplastic inflammatory conditions like chronic infections (tuberculosis, Helicobacter pylori), autoimmune diseases such as rheumatoid arthritis, multiple sclerosis, myasthenia gravis, and inflammatory bowel disease [4]. However, TLSs have been identified in the microenvironment of different tumor models, including melanoma and lung, colorectal, and breast cancer [5,6,7].

Remarkably, the presence of TLSs has been shown to be independent of tumor mutational burden, which can influence the immune response and TILs in several tumor entities [8].

## 2. Materials and Methods

A comprehensive literature assessment was performed by querying the PubMed/Medline, Scopus, and EMBASE databases which were systematically searched from their inception to August 2023. The authors reviewed retrospective studies, meta-analyses, reviews, and basic studies that encompassed the topic of tertiary lymphoid structures in cancer and breast cancer. The search terms used were: “tertiary lymphoid structures”; “cancer”; “breast cancer”; “predictive value”; “prognostic value”; “pathological complete response”; “immunotherapy”; and “immune microenvironment”. The articles were selected by the authors based on their relevance for the development of a narrative review.

## 3. Results

### 3.1. Organization of TLSs

**Figure 1:**
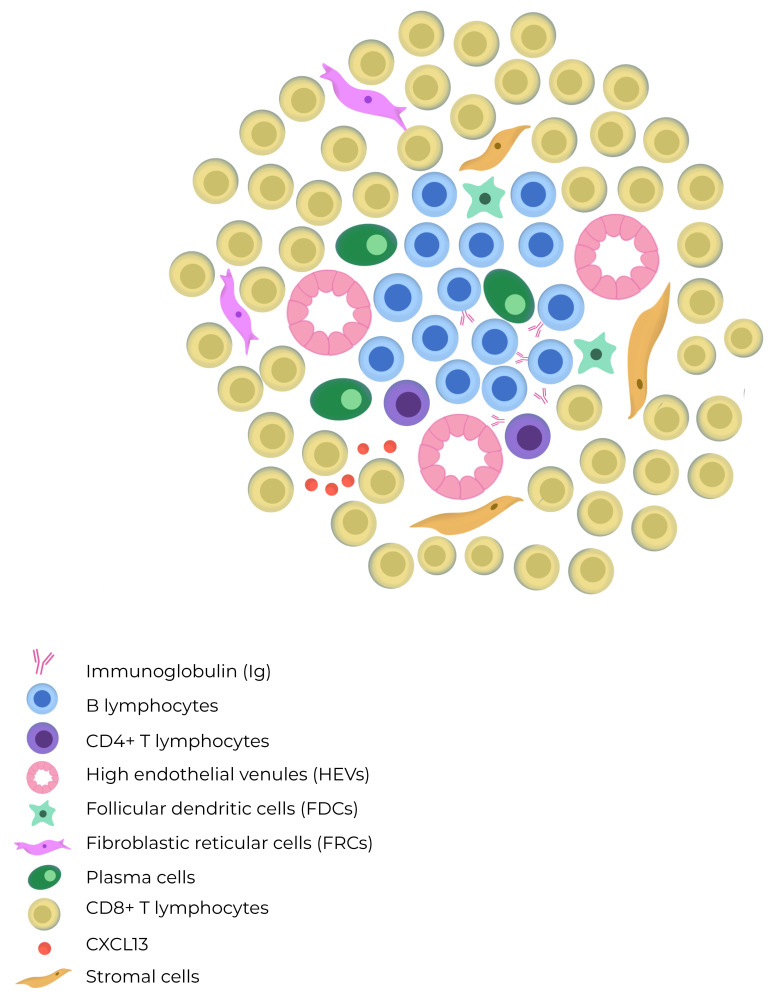
The composition of a tertiary lymphoid structure includes B cells which are mostly intermingled, but occasionally they form distinct B cell and T cell compartments resembling those found in lymph nodes. TLSs lack a capsule and exhibit high endothelial venules (HEVs) in their architecture. The T cell zone contains mature dendritic cells and fibroblastic reticular cells, whereas the B cell zone has a germinal center with plasma cells, macrophages, and follicular dendritic cells.

The mechanisms involved in the induction of TLSs remain a subject of debate. Some aspects of TLS organization are inherently linked to the development of lymph nodes and secondary lymphoid structures (SLSs) during embryogenesis. Pimienta et al. showed in their analysis that specific chemokines, such as CCL19, CCL21, and CXCL13, secreted by the surrounding mesenchyme, also play a role in attracting Lymphoid-tissue-inducing (LTi) cells and lymphocyte subsets that populate the developing lymph node [9]. Then, CCR7, which is a receptor present on subsets of T cell and dendritic cells (DCs), prompts their activation through CCL19 and CCL21 signaling. CCR7 activation is crucial for recruiting memory T cells and DCs during immune responses. CXCL13 facilitates the influx of migratory B cell that express the CXCR5 receptor [10]. Also, circulating immune cells such as B, T, or dendritic cells can act as LTi cells in response to chemokines secreted by injured tissue. LTα promotes HEV growth and activation of follicular helper T cells (Tfh), which might represent circulating counterparts of follicular dendritic cells (FDCs). Furthermore, the architecture of TLSs typically features a zone of T cells adjacent to a B cell follicle, resembling the structure of SLSs and mucosa-associated lymphoid tissues [9]. TLSs are characterized by loosely organized structures containing HEV-like structures, which differ from the classic HEVs typically found in peripheral lymph nodes [11]. These specialized blood vessels contribute to the migration dynamics of central and naïve memory T cell. They also support naïve B cell structurally, aiding the effects of chemotactic cytokines such as CCL19, CCL21, CXCL12, and CXCL13. In Figure 1, the cellular composition of a tertiary lymphoid structure is illustrated.

TLSs can adopt diverse histoarchitectural patterns, comprising a B-cell compartment that incorporates CD23+ germinal center B cell (GC-B) and peripheral naïve, plasma (PC), and memory B cell. In close proximity, Tfh cells are the predominant cell type in the T-cell compartment of TLSs, suggesting their role as regulators of the B lymphocyte lineage. Other essential cell subpopulations found within TLSs include fibroblastic reticular cells (FRCs), which play a specific regulatory role in T-cell development, and dendritic cell (DC) subpopulations that may vary within TLS compartments. These DC subpopulations include dendritic cell lysosomal-associated membrane protein (DC-LAMP) and CD21+ follicular dendritic cells (FDCs), which are found in the T and B cell regions, respectively [12]. One characteristic of TLSs is their plasticity; they can form temporarily and then disappear when the antigen is removed. The location of lymphoid aggregates is predetermined by the expression of the lymphotoxin β receptor (LTβR) on endothelial cells [13,14]. Meylan et al. showed that in TLS-associated tumors, IgG- and IgA-producing plasma cells (PCs) spread to tumor beds along fibroblastic pathways. Tumors that express TLS, in turn, exhibit IgG-producing PCs and IgG-stained apoptotic malignant cells, indicating potential antitumor activity [15]. Evidence has shown that the antibody subtype produced by B lymphocytes plays a crucial role in the immune response. IgG+ and IgA+ PCs are found within TLSs and are also present near tumors, alongside CXCL-12 and COL1 positive fibroblasts. Additionally, tumors exhibiting TLSs are coated with IgG antibodies and have augmented proapoptotic signaling [16]. Furthermore, Harris et al. have recently demonstrated that clonal expansion towards IgG isotype production is a favorable prognostic factor in breast cancer for disease-free survival, particularly in triple-negative breast cancer (TNBC) [17].

### 3.2. Role in Solid Tumors

TLSs have been predominantly linked to a favorable clinical outcome in patients with various types of solid tumors, such as hepatocarcinoma and colon cancer, where they have demonstrated an improvement in recurrence-free survival (RFS) [18,19,20]. Similarly, in melanoma, TLSs have been associated with improved overall survival and recurrence-free survival in patients treated with ICIs. One possible explanation for these findings is that TLSs are associated with a higher density of CD8+ T lymphocytes that infiltrate the tumor with an activated and cytotoxic immune signature [21,22].

In Table 1 we summarize selected studies studying the prognostic role of TLSs in different tumor models.

The case of soft tissue sarcomas is particularly noteworthy due to their typical classification as non-immune-responsive tumors. However, in recent exploratory analyses, an association was established between intratumoral plasma cell (PC) abundance and progression-free survival in patients who were treated with pembrolizumab and cyclophosphamide [29,30].

### 3.3. TLS in Breast Cancer

Immunological profiling of breast tumors has provided valuable insights into potential immune evasion mechanisms in breast cancer and has revealed unique aspects of the tumor microenvironment (TME) [9].

A significant area of interest in breast cancer research involves targeting immune checkpoint molecules, particularly in the triple-negative subtype, where treatment options remain limited. The expression of immune checkpoint signals has been associated with the presence of TILs and TLSs, although there is notable heterogeneity, even within the same biological tumor subtypes. TLSs are predominantly found in the stromal region of approximately 60% of breast cancers [5]. The infiltration of immune cells in breast tumors has proven to be both predictive and prognostic. Tumors with higher levels of TILs were described as responding more favorably to various treatments, such as ICIs, chemotherapy, and radiation, compared with tumors with low TILs [31]. Despite the growing interest in immune checkpoint blockade in breast cancer, the response rates achieved with single-agent therapy have been relatively low in the metastatic setting, indicating the presence of intrinsic or innate resistance mechanisms [30]. GuTrantien et al. devised an 8-gene signature to reflect the presence of TLSs [32]. This signature was found to be prognostic in breast cancer patients undergoing surgical resection with or without neoadjuvant chemotherapy. In particular, CXCL13 expression was found to have the closest association with the prognostic signature in this cohort. Moreover, this cytokine has also been found to allow identification of TLSs in colorectal cancer and soft tissue sarcoma.

Another genomic platform evaluated the prognostic role of 12 genes which encode chemokines (12-CK) associated with immunity and inflammation in different tumor models [33]. In the breast cancer subgroup, authors described that basal and HER2-enriched tumors, and a high calculated score of chemokine-gene expression was associated with higher DFS and OS. By molecular subtypes, patients with the basal subtype and HER2 obtained a survival benefit by having a high expression of the 12-CK genes.

Wang et al. [34] conducted a meta-analysis involving 15 studies with 3898 patients. Their combined analysis revealed that the presence of TLSs was associated with improved disease-free survival and overall survival. Additionally, TLS presence was positively correlated with early tumor TNM stage and high tumor-infiltrating lymphocytes. It was also associated with human epidermal growth factor receptor 2 and Ki-67, but inversely correlated with the status of estrogen and progesterone receptors. The study also found that the tumor immune microenvironment was more favorable in the high-TLS signature group, leading to better survival outcomes for breast cancer patients.

Immune checkpoint blockade has yielded remarkable and enduring responses across various cancer types. Nevertheless, not all patients exhibit favorable responses, and the currently available biomarkers (such as PDL1 expression, tumor-infiltrating lymphocytes, and tumor mutational burden) do not consistently provide reliable predictions of treatment outcomes. Consequently, there exists an urgent need for novel and more specific biomarkers to guide the clinical practice [35]. Tertiary lymphoid structures (TLSs) have long been acknowledged as prognostic markers for enhancing patient outcomes. Recently, mature TLSs have been identified as predictors of successful outcomes in patients treated with immune checkpoint inhibitors. This predictive capability likely arises from their role in eliciting immunogenic cell death, leading to the release of neoantigens [36]. The findings from a study by Solinas et al. [9]. revealed that TLSs in breast cancer were infiltrated with cells expressing PD-L1, PD-L2, LAG3, and TIM3, suggesting that TLSs represent important sites of immune activation and regulation. However, it is important to note that although information from preclinical trials suggests that TLSs could serve as valuable predictors of immunotherapy effectiveness, further clinical and translational investigations are still necessary [37,38].

In Table 2, we summarize selected studies which explore the prognostic and predictive role of TLSs in breast cancer, highlighting favorable outcomes in DFS, OS, and pCR.

#### 3.3.1. HER2-Positive Tumors

In HER2+ tumors, the presence of macrophage infiltration has been associated with the presence of TLSs. Additionally, a strong correlation was reported between the extension of the ductal carcinoma in situ (DCIS) component of an invasive lesion and the identification of TLSs [43]. According to the findings of the retrospective study by Xia Liu, which involved 248 samples of both HER2-positive and HER2-negative breast cancer, it was demonstrated that TLSs were associated with a prolonged PFS. Importantly, the authors noted that TLSs were not correlated with the presence of TILs, supporting the characterization of TLSs as an independent prognostic biomarker [2]. Based on these findings, it is possible to speculate that increased HER2 protein expression may contribute to a pro-immunogenic environment attracting lymphocytes to the tissue and promoting TLS formation. Another potential explanation could be related to the frequent presence of comedonecrosis in HER2+ DCIS, which may lead to an increased infiltration of macrophages. Macrophages serve as antigen-presenting cells in the antitumor immune response, potentially contributing to the development of TLSs.

Gu-Trantian and colleagues characterized the way that CXCL13-producing follicular helper T (TFH) cells were associated with better disease-free survival (DFS) and complete pathological response in patients with localized HER2-positive tumors [32,44]. Xia Liu and colleagues also showed an association with TLS presence and better prognosis in a cohort of 55 patients with HER2+ breast cancer. This was not associated with other ICI-associated predictive biomarkers, such as the level of TILs [2].

The HER2DX assay developed and validated by Prat et al. shows that HER2-positive tumors with a higher immune profile have better rates of complete pathological response after neoadjuvant anti-HER2-based chemotherapy. HER2DX is based on four distinct gene signatures encompassing 27 genes, capturing diverse biological processes, including immune infiltration, tumor cell proliferation, luminal differentiation, and HER2 amplicon expression. It was observed that tumors with increased levels of CD4+, CD8+, CD20+ s-TILs, and CD20+ intratumoral TILs were independently associated with a higher probability of achieving a pathological complete response (*p* = 0.03) [45].

#### 3.3.2. HR-Positive Breast Tumors

Traditionally, luminal subtype tumors are considered to have a less inflammatory microenvironment compared with their triple-negative or HER2-positive counterparts. These tumors typically exhibit high FOXP1 expression and low levels of TLSs [46]. This microenvironment can be explained by the inverse correlation between increased lymphocytic infiltration and the expression of estrogen receptor (ER) and/or progesterone receptor (PR) [47]. Higher FOXP1 expression is observed in ER-positive breast cancers, which are associated with reduced TILs and TLSs. Additionally, FOXP1-high tumors show elevated levels of IL10 and TGFβ, creating an immunosuppressive milieu [48]. However, the initial outcomes of the first pre-specified interim analysis of the KEYNOTE-756 randomized and double-blind phase III trial have recently been disclosed. The association of ICI treatment with higher pCR in a high-risk ER+ subgroup supports the necessity of adequately identifying which biomarkers can better explain the treatment response to these agents [49].

Another intriguing aspect concerning HR-positive tumors is that prior treatment with CDK inhibitors (CDKi) in the metastatic setting has shown the potential to stimulate heightened immune responses. In preclinical models, CDK4/6 inhibitors were associated with enhanced tumor antigen presentation, reduced proliferation of regulatory T cells (Tregs), and a lower expression of immune inhibitory receptors, such as PD-1 [50].

#### 3.3.3. Triple-Negative Breast Tumors

Tumors categorized in the triple-negative breast cancer (TNBC) subgroup exhibit TLSs in 83.7% of cases [51]. Within this subgroup, the level of FOXP1 holds significance as it regulates the chemokine CXCL13, a B-cell chemoattractant. Similar findings were reported by Song et al., who analyzed a cohort of 108 TNBC patients who underwent neoadjuvant chemotherapy. They found correlations between TLS density, CD8+ and CD20+ lymphocytes and CXCL13 expression, and pathological complete response (pCR) [5]. In the metastatic setting, PD-L1 expression is a predictive factor for ICI response in this patient subgroup [52]. Moreover, we are aware that neoadjuvant immunotherapy is now considered the standard treatment approach for TNBC. Nevertheless, further research is still needed within this subgroup to assess the prognostic value of TLSs to understand their role as an independent biomarker. According to the results from the study conducted by Vanhersecke and colleagues [22], which evaluated the role of TLSs in samples with positive and negative PD-L1 tumors, according to a cutoff value of 1% for tumor proportion score, patients with TLS-positive tumors had response rates of 69.2% and 40.3%, considering PD-L1-positive and PD-L1-negative tumors, respectively. In turn, patients with TLS-negative tumors had significantly lower response rates, with these being 35.6% and 14.1%, respectively. In this study, the proportion of PD-L1-positive tumors was comparable between those with a high TLS density (22.4%) and those with low TLS density or no TLSs (21.1%). Remarkably, regardless of the PD-L1 expression status, patients with TLS-positive tumors demonstrated superior outcomes.

### 3.4. Metastatic Breast Cancer

The tumor microenvironment of the primary tumor differs from those of metastases, due to factors such as the immunosuppressive stroma. A study assessed the correlation of TLS presence in the primary tumor and metastases (in 101 surgically removed specimens) [53]. Tertiary lymphoid structures were present in all primary breast cancers, and surgical pieces of lung, liver, brain, and ovarian metastases were evaluated. In metastases, tertiary lymphoid structures were only found in lung and liver metastases. Primary tumors with tertiary lymphoid structures exhibited significantly higher levels of tumor-infiltrating lymphocytes than those without tertiary lymphoid structures, across all metastatic sites.

### 3.5. Diagnosis of TLSs

Diagnosing TLSs in the tumor microenvironment is a complex task due to various factors, including the heterogeneity of TLSs and the tumoral microenvironment. For example, the evaluation of PD-L1 expression, a commonly used biomarker, is susceptible to high inter-observer variability due to the use of different antibodies, platforms, and scoring systems [54,55]. When selecting tissue for TLS diagnosis, it is crucial to acknowledge that these structures exhibit spatial variations within tumor tissues and may not be fully represented when in small-needle biopsy samples or tissue microarrays. This challenge is particularly significant in breast cancer patients, as they often undergo neoadjuvant systemic therapy following an initial diagnosis with a core needle biopsy, limiting the availability of tissue samples to adequately assess the presence of TLSs [2,56].

TLS identification methods can vary based on the tumor type and specific research goals. Commonly employed techniques for quantifying TLSs include recognizing immune cell markers and immunohistochemical labeling of components such as high endothelial venules. Staining for CD21 and CD23, indicative of the presence of follicular dendritic cells (FDCs), is frequently used for TLS identification. Additionally, the co-localization of CD3+ T cells and CD20+ B cell represents another reported technique [57].

Another method for identifying TLSs involves utilizing gene or chemokine signatures in immuno-histochemistry. TLSs can be detected by analyzing the expression of specific genes or chemokines associated with their development [58].

Lastly, digital and computational pathology, integrating deep learning and artificial intelligence, can prove instrumental for both identifying and quantifying TLSs [59]. Multiplex fluorescent immunohistochemistry/immunofluorescence (mIHC/IF) has demonstrated superior performance compared with immunohistochemistry. These advanced methods enable simultaneous assessment of multiple targets, detection of cell densities and sub-populations, estimation of the functional states of the immune infiltrate, and characterization of spatial organization via analyzing cell–cell interactions and distribution across various regions of interest and tissue compartments [36]. Digital imaging enhances reproducibility in the analysis of complex spatial protein profiles in tumor tissues.

Although immunological signatures offer valuable insights, their limitations include the absence of spatial information and potential dominance by the most abundant cell population, which limits their accuracy. Additionally, the assessment of TILs through hematoxylin and eosin staining presents challenges, as this involves a mass measurement of stromal lymphocytic infiltration and is susceptible to high interobserver variability [60]. One drawback of this diagnostic approach is that not all tumor microenvironment components are easily detected via immunohistochemistry, such as intracellular cytokines reflecting the functional state of the tumor milieu. Consequently, techniques combining tissue biomarker characterization and in situ transcriptional profiling have been developed [61,62]. As exemplified by the results of the PEMBROSARC trial [29,63], there is a hypothesis that the spatial distribution, rather than the density, of B cell within tumors may better predict treatment response. Tumors exhibiting a greater dispersion of lymphoid structures and a higher density of CD8-T cells may have a better prognosis.

It is crucial to note that the significance extends beyond the inflammatory component of the primary tumor to include the presence of TLSs in metastases. Notably, lung metastases show an association with improved overall survival [64,65].

### 3.6. Single-Cell scRNA-Seq Evidence on TLSs in Breast Cancer

Single-cell sequencing has emerged as a novel tool for studying the tumor microenvironment. The development of single-cell RNA sequencing (scRNA-Seq) allows us to dissect gene expression at the single-cell level and identify new biomarkers with improved performance. This type of analysis can provide evidence of the effect of TLSs in the tumor microenvironment. Wang Q. et al. [66] investigated this through the search for 12-CK cytokines. They could predict the presence of TLSs as “High” and “Low.”

Another key utility of scRNA-seq is the study of resistance to immunotherapy. The potential culprit could be cancer stemness. Zhang et al. [67] collected and analyzed publicly available scRNA-Seq datasets from patients treated with immune checkpoint inhibitors (ICIs) to elucidate the association between cancer stemness and ICI response. They developed a new tool for evaluating stemness signature (Stem.Sig). A negative association was found between stemness signature and anti-tumor immunity, whereas positive correlations were detected between intratumoral heterogeneity and tumor mutational burden (TMB). This could be a promising solution for patient selection for immunotherapy treatment.

### 3.7. TLS as a Predictive Factor for Immunotherapy Response

Since 2020, several studies have identified an association between mature TLSs and improved response to immunotherapy across various cancer types. Clubb et al. [68] discovered a synergistic interaction between TLS formation and anti-PD-1/PD-L1 immunotherapy in head and neck cancer. This interaction is linked to the role of TLS in maintaining the immune response microenvironment, suggesting that the effectiveness of immunotherapy could be enhanced by inducing TLS formation or increasing TLS maturity. Zhong et al. [69] used high TLS density as a criterion to predict the response to combined anti-PD-1 immunotherapy with sorafenib in HCC patients with portal vein thrombosis. They found a strong predictive performance, indicating that TLSs hold value as a predictive indicator for immunotherapy response. Cabrita et al. [28] evaluated this aspect in melanoma patient samples. The TLS signature (among three other immunologic signatures) demonstrated the best predictive performance for immunotherapy response. The TLS signature also remained independent of the tumor mutational burden in the anti-PD1-treated cohort.

In breast cancer, Wang L. et al. [70] assessed the effect of the TLS-associated gene signature based on The Cancer Genome Atlas (TCGA) dataset in breast cancer patients. Comprehensive results showed that patients with a high TLS signature benefit from immunotherapy, making it a promising biomarker to distinguish prognosis and the immunological microenvironment in breast cancer.

### 3.8. TLS Manipulation as Therapeutic Opportunity

TLSs are not merely a surrogate indicator of an active immune response; rather, they are believed to actively modulate the anti-tumor immune activity. In addition to serving as markers of responses to immunotherapy, TLSs have the potential to act as therapeutic agents in promoting anti-tumor immune responses. This is achieved through the recruitment of lymphocytes, control of tumor growth, and the induction of a long-lasting humoral anti-tumor immune response [58,71], akin to therapies involving chimeric antigen receptor T (CAR-T) cells [72].

Furthermore, in mouse models, it has been demonstrated that the administration of CCL21, CXCL13, or LTα alone can induce the formation of TLSs [9]. The induction of de novo TLS formation holds significant therapeutic promise in overcoming inherent immune inhibitory mechanisms within the tumor microenvironment. This approach can potentially convert immunologically “cold” tumors, which do not respond to immunotherapy, into “hot” tumors that are susceptible to treatment [73]. Nonetheless, these findings should be considered only as hypothesis-generating and further research is strongly needed to determine whether this biomarker may be evaluated for microenvironment-modulating therapies.

## 4. Conclusions

In conclusion, the landscape of cancer immunotherapy response prediction is undergoing a transformative shift, with TLSs emerging as a compelling and potentially more robust biomarker compared with the extensively studied PD-L1 expression and TILs. The ongoing investigation of TLSs as a treatment response predictor signifies a dynamic frontier in precision immuno-oncology, promising to enhance patient outcomes.

The existing evidence strongly emphasizes the remarkable ability of TLSs to distinguish between responders to anti-PD1/PDL1 therapy, offering a pivotal mechanism for effectively modulating the tumor microenvironment in cancer immunotherapies. Moreover, the induction of TLSs opens an exciting new therapeutic avenue in the battle against cancer, calling for the implementation of standardized pathology methodologies to accurately detect and diagnose this intriguing biomarker.

Although the retrospective evidence demonstrates TLSs have better predictive value than PD-L1 in early breast cancer patients treated with ICIs, it also underscores the need for prospective studies to validate and fully comprehend the impact of spatial distribution on prognosis. As we navigate the frontier of precision medicine, ongoing research in this field promises to revolutionize cancer care, paving the way for tailored therapies and unprecedented advancements in immuno-oncology. The exploration of TLSs as a promising avenue requires collaborative endeavors within the scientific community to determine whether it is possible to identify a better biomarker than PD-L1.

## Figures and Tables

**Table 1:** Studies showing the prognostic and predictive value of TLS response to immunotherapy in various solid tumor models.

Author	Year	Tumor Model	Sample Size	Tipo	Recognition of TLS	Findings
Rutao Li [23]	2022	Esophagus	185 primary ESCC treated by surgical resection	Retrospective	IHC for CD45+, CD20+ B, CD4+ CD8+ T cells, CD11c+ DC	Better DFS (*p* = 0.0130) and OS (*p* = 0.0164).
Nana Zhang [12]	2020	Gastric adenocarcinoma	180 gastric adenocarcinoma samples (surgery)	Retrospective	IHC staining and MECA-79 + (HEV)	Better OS (*p* = 0.007)
Qunxing Li [24]	2020	Oral Squamous cell carcinoma	168	Retrospective	Multiplex IHC (CD3+ T cells, CD20+ B cell, PNAd+ HEV DC-LAMP + (LAMP3)	Independent prognostic factor for 5-year OS rate (HR = 3.78) and RFS rate (HR = 3.29)
Sho Wakasu [5]	2022	Lung adeno carcinoma	218	Retrospective	The overlap of T-cell zone and B-cell zone	Better OS [HR] = 0.17, (*p* = 0.0220) and DFS (HR = 0.54, *p* = 0.0436).
Nanda Horeweg [6]	2022	Endometrial adenocarcinoma	411 (All included patients from the PORTEC-3 study)	Retrospective	scRNA-seq of B-cells to establish the presence of cycling/germinal center B-cells and antibody-secreting B-cells	Better RFS, independent of clinicopathological and molecular factors
Di Caro et al. [7]	2014	Colorectal	351 stage II and III colorectal cancer without any sign of metastatic disease	Retrospective	IHC (CD3, CD20, PNAd, Lyve-1, CD21, α-smooth muscle actin and CXCL13 and CCL21	Better RFS (relapse; *p* = 0.03)
Posch et al. [19]	2018	Colo rectal	109 patients with stage II/III nmCRC	Retrospective	NR	Better RR of recurrence. (HR for low TLS = 3.99, 95% CI: 1.30–12.20, *p* = 0.015
Meshcheryakova et al. [20]	2014	Colo rectal	65 metastatic colorectal cancer in the liver	Retrospective	IHQ (CD45, CD20, AID, IgM, CD138, and CD68)	Better RFS (*p* < 0.001)
Julien Calderaro [18]	2019	HCC	273 patients with HCC treated by surgical resection	Retrospective	Pathological review NR	Lower risk of early relapse (<2 years after surgery, hazard ratio 0.46, *p* = 0.005).
Germain C. [25]	2014	Lung Cancer	74 untreated patients with early-stage NSCLC	Retrospective	Immunohistochemistry. Characterization of CD20 B-cell subsets by flow cytometry.	Better OS Better DSS
Van Dijk [26]	2021	Urothelial cancer	31 cystectomy specimens obtained from NABUCCO	Retrospective	Multiplex immunofluorescence (CD3, CD8, FoxP3, CD68, CD20, PanCK, DAPI)	Better RFS (*p* = 0.0097)
Lynch et al. [27]	2021	Melanoma (metastases)	64 patients	Retrospective	Multiplex immunofluorescence.	Better OS (HR 0.51, *p* = 0.04)
Cabrita et al. [28]	2020	Melanoma	177	Retrospective	IHC Anti-CD20 AntiCXCR5 and Anti-CXCL13	Better 5 year OS (*p* = 0.006)
Italiano et al. [29]	2022	Sarcomas	30 samples	Multicohort phase 2 study of pembrolizumab combined with low-dose cyclophosphamide	NR	Better 6-month NPR (non-progression rate) NPR = 40% ORR 30%
Maxime Meylan [15]	2022	Renal cell cancer	Primary tumors (*n* = 130) from three cohorts of treatment-naïve patients with ccRCC	Retrospective	Visium 10X spatial transcriptomics technique that allowed both quantification and localization of B cell-specific gene expression.	Better PFS and response to ICI

**Table 2:** Studies demonstrating the prognostic value of TLS in different subtypes of breast cancer.

Author	Breast Cancer Subtype	Type	N	Year of Publication	Recognition of TLS	Findings
Lee et al. [39]	TNBC localized	Retrospective	769	2016	IHC for MECA-79 and CD31	Better DFS Better OS
Xia Liu [2]	HER2 + and negative breast cancer	Retrospective	248	2017	IHC CD3, CD20, and CD23	Better DFS (log-rank = 4.054, *p* = 0.044)
Song [40]	TNBC localized	Retrospective	108 TNBC patients treated with neoadjuvant chemotherapy	2017	IHC for MECA79, CD3, CD8, and CD20 and Nanostring analysis of CXCL13	Better pCR Better DFS
Bin Wang [34]	BC	Systematic Review and Meta-Analysis (PRISMA) criteria	15 studies with a total of 3898 patients	2022	NR	Better DFS (HR = 0.61, *p* < 0.05) OS (HR = 1.66, *p*< 0.001)
Kezhen Li [41]	Localized breast cancer	Retrospective	242 patients with localized primary BC (confirmed by surgery)	2023	NR	Better 3-year DFS
Noel et al. [42]	TNBC (27) & HER2+ (21)	Retrospective	48	2021	IHC CD3/CD20	Better DFS (*p* value = 0.001)
